# The ventromedial hypothalamic nucleus: watchdog of whole-body glucose homeostasis

**DOI:** 10.1186/s13578-022-00799-2

**Published:** 2022-05-26

**Authors:** Longlong Tu, Makoto Fukuda, Qingchun Tong, Yong Xu

**Affiliations:** 1 USDA/ARS Children’s Nutrition Research Center, Department of Pediatrics, Baylor College of Medicine, 1100 Bates Street #8066, Houston, TX 77030 USA; 2 Brown Foundation Institute of Molecular Medicine, University of Texas Health Science Center at Houston, Houston, TX 77030 USA; 3 Department of Molecular and Cellular Biology, Baylor College of Medicine, Houston, TX 77030 USA

**Keywords:** VMH, Glucose sensing, Whole-body glucose homeostasis, Counterregulatory response, Hypoglycemia, Diabetes, Sex difference

## Abstract

The brain, particularly the ventromedial hypothalamic nucleus (VMH), has been long known for its involvement in glucose sensing and whole-body glucose homeostasis. However, it is still not fully understood how the brain detects and responds to the changes in the circulating glucose levels, as well as brain-body coordinated control of glucose homeostasis. In this review, we address the growing evidence implicating the brain in glucose homeostasis, especially in the contexts of hypoglycemia and diabetes. In addition to neurons, we emphasize the potential roles played by non-neuronal cells, as well as extracellular matrix in the hypothalamus in whole-body glucose homeostasis. Further, we review the ionic mechanisms by which glucose-sensing neurons sense fluctuations of ambient glucose levels. We also introduce the significant implications of heterogeneous neurons in the VMH upon glucose sensing and whole-body glucose homeostasis, in which sex difference is also addressed. Meanwhile, research gaps have also been identified, which necessities further mechanistic studies in future.

## Introduction

Glucose provides the most essential energy source for neurons and non-neuronal cells (e.g., astrocytes and tanycytes) in the brain. Glucose levels therefore need to be regulated and maintained within a narrow physiological range, which implicates a number of neural and hormonal signals derived from glucose sensors. These glucose sensors are widely distributed in both the central nervous system (CNS) and the periphery (e.g., the portal/mesenteric vein, the endocrine pancreas, taste buds of the togue and the gut) [1]. Imbalance in whole-body glucose homeostasis is closely linked with metabolic complications such as diabetes and obesity, which nowadays have become growing global health concerns [2]. Thus, understanding cellular and molecular mechanisms of glucose sensing, as well as whole-body glucose homeostasis is of paramount importance for the development of new strategies to prevent and treat metabolic disorders.

The outbreak of COVID-19 pandemic reminds that patients with diabetes and/or obesity are more likely to develop severe symptoms, concomitant with a high mortality rate [3]. It is worthy to note that fasting glucose levels have been used to predict the 28-day mortality in patients with COVID-19 even without previous diagnosis of diabetes [4]. Moreover, COVID-19 is also associated with aberrant glucose metabolic disorders [5]. All the evidence suggests that imbalanced glucose might be a risky factor exacerbating symptoms and even morality of COVID-19. However, the link between glucose homeostasis and COVID-19 is still not clear, and additional investigations is still an important priority.

In 1849 Claude Bernard made the original observation that the brain is able to detect hypoglycaemia, and instigate a suitable counterregulatory response (CRR) to restore euglycemia [6]. In the year of 1953, John Mayers proposed that there are cells in the hypothalamus to monitor fluctuations of circulating glucose levels and translate into chemical or electrical responses that control food intake [7]. Glucose-sensing neurons were then discovered in the brain of cats and dogs by two different groups in the early 1960s using in vivo extracellular recordings [8, 9]. More specifically, these two studies have demonstrated that both food intake and infusion of glucose increase the activity in half of recorded neurons in the ventromedial hypothalamic nucleus (VMH), but decrease the activity in half of monitored neurons in the lateral hypothalamus (LH) [8, 9]. By contrast, fasting and insulin induce a more prominent neuronal activity within the LH than the VMH [8, 9]. These findings, for the first time, indicate that a high glucose level activates a subpopulation of neurons in the VMH, and yet inhibits a distinct cluster of neurons in the LH, while a low glucose level acts in an opposite manner [8, 9].

**Fig. 1:**
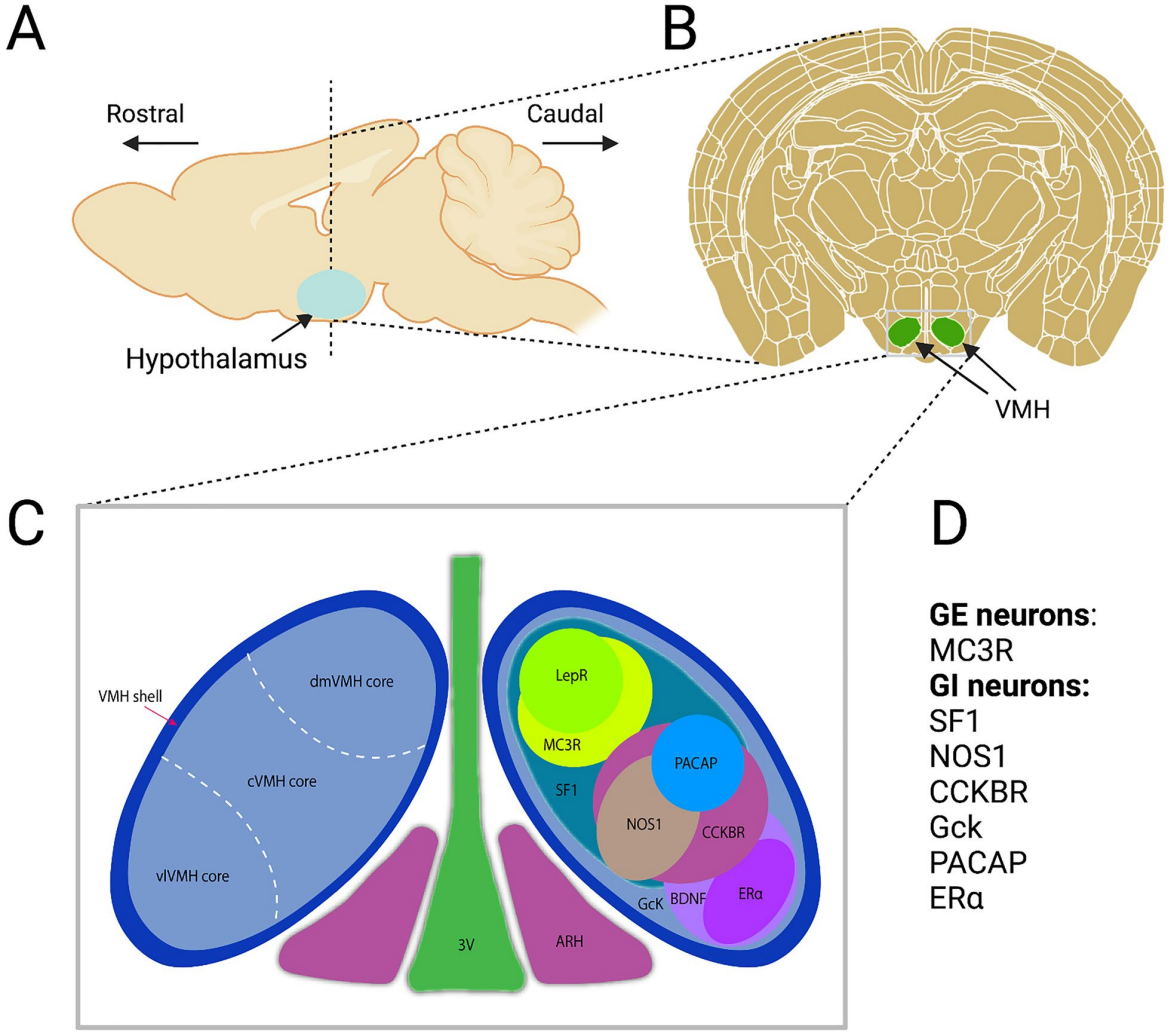
Schematic illustration of identified neuronal clusters in the VMH involved in glucose homeostasis and other homeostatic responses. **A** The location of the hypothalamus in the brain in a sagittal section. **B** The location of the VMH in the hypothalamus in a coronal section. **C** Schematic presentations of anatomical structure and patterns of genes highly expressed in the VMH. The VMH contains a core and a shell region. The core region is comprised of three subdivisions (i.e., dmVMH, cVMH and vlVMH). Most neurons in the core region are glutamatergic, yet few of them are GABAergic. The majority of neurons in the shell are GABAergic. **D** GE vs. GI neurons in the VMH. GE and GI neurons are classified based on the effect of activation of each specific cluster of neurons in the VMH. For instance, some SF1 neurons are GE, and some are GI neurons. However, activation of SF1 neurons increases systemic blood glucose, we therefore classify SF1 neurons into GI neurons. 3 V, third ventricle. ARH, arcuate nucleus of hypothalamus. BDNF, brain-derived neurotrophic factor. CCKBR, cholecystokinin receptor B. ERα, Estrogen receptor-α. GcK, glucokinase. LepRs, leptin receptors. MC3R, melanocortin 3 receptor. NOS1, nitric oxide synthase 1. PACAP, pituitary adenylate cyclase-activating peptide. SF1, steroidogenic factor 1. VMH, ventromedial hypothalamic nucleus. These schematic diagrams were generated based on articles from Ref. [2]

Since then, the brain has been regarded as a key regulator of glucose homeostasis, in which the hypothalamus, comprised of a plethora of distinct nuclei, is one of the most critical centres mediating glycemic control [10, 11]. Among those distinct hypothalamic nuclei, the VMH has been recognized as one of the most essential sites for glucose sensing and whole-body glucose homeostasis [12–15] (Fig. 1). Emerging studies reveal that dysfunction of glucoregulatory circuits in the brain, and impaired coordination between the brain and periphery occur in certain metabolic complications (see the reviews Ref. [16, 17].) It has been speculated that there is a significant but not fully elucidated role played by the brain in the pathogenesis of type 2 diabetes (T2D) and obesity [18, 19]. Indeed, a multitude of studies focusing on the brain give rise to remarkable progress in improving glucose homeostasis in various rodent models of diabetes [17, 20]. More importantly, this might lead to strategies for development of new remedies of diabetes and obesity: whether manipulation of the brain (e.g., neurons in the VMH or elsewhere in the brain) can be used to improve glycemic control in people with diabetes and/or obesity [16, 17]. Certainly, the studies of the brain in glucose homeostasis necessitates novel technologies enabling to manipulate specific neurons or other types of cells in the context of glucoregulation in vivo. Recent advance in neuroscience tools, including sophisticated mouse genetics, optogenetic and chemogenetic manipulation, calcium indicator and single-cell sequencing, etc., makes it achievable, and more importantly, reliable.

## Central regulation of glucose homeostasis

The conventional concept of glycemic control is islet-centric—that coordination of insulin, glucagon and other counterregulatory hormones (i.e., cortisol and epinephrine) is able to regulate blood glucose level within a narrow range under physiological conditions (e.g., fasting, feeding) [11, 17]. However, the role of the brain in glucose homeostasis cannot be neglected.

It is possible that the brain plays a minor role in whole-body glucose homeostasis under physiological conditions. Stated differently, pancreatic islets are able to tightly control the blood glucose even without input from the brain (e.g., postprandial glucose regulation). Indeed, the brain is able to regulate islet hormones secretion like insulin; it is however still unknown whether the brain is also implicated in the regulation of insulin during feeding [21]. Another possibility is that the brain works cooperatively with islets and even other peripheral tissues (e.g., liver, adipose) to regulate glucose homeostasis [11]. For example, peripheral glucose sensors (e.g., glucose-sensing neurons in the hepatic portal vein) sense the circulating blood glucose levels and convey this signal to the brain, such that the brain regulate blood glucose mainly via autonomic nervous system [22]. In this scenario, astrocytes may also be engaged in the process of glucose detection [23]. Unlike neurons sitting behind the blood brain barrier (BBB), astrocytes are able to sense circulating glucose level via end-foot process that enwrap capillaries and pericytes of the BBB [24]. Therefore, neurons in the brain can receive the signal of blood glucose via interaction with astrocytes. Indeed, there is no consensus upon what the brain exactly does under physiological conditions. However, the cooperation between the brain and endocrine islets is of essential importance for pathogenesis of diabetes/obesity [11, 17]. Exhaustive studies, not only restricted to the VMH, but also other brain regions, as well as the interaction with peripheral tissues, are therefore warranted.

The most widely accepted pathogenesis of T2D is that increased insulin secretion fails to compensate for insulin resistance in the periphery [25, 26]. Thus, drugs enhance insulin production and/or increase insulin sensitivity are being developed to treat diabetes. On top of glucose, insulin also exerts widespread and varied actions in the brain including whole-body glucose homeostasis. Current evidence suggests that the brain contributes to whole-body glucose homeostasis partially via insulin-dependent mechanisms [27]. The brain was once thought as insulin-insensitive organ as glucose uptake in the brain is insulin-independent [28]. However, subsequent studies reveal that insulin receptors are densely populated throughout the brain including the VMH, where a higher percentage of glucose-sensing neurons express insulin receptor than non-glucose-sensing neurons [29, 30]. The available evidence indicates that insulin directly acts on the hypothalamus to modulate hepatic glucose production, thereby whole-body glucose balance [31, 32]. Of note, it has been reported that insulin resistance, lack of insulin or hypoglycemia associated automimic failure would affect glucose sensitivity of glucose-sensing neurons, thereby whole-body glucose homeostasis (see the review Ref. [33]). Obviously, the major glucoregulatory effect of insulin is to promote glucose utilization in the peripheral tissues (e.g., liver, skeletal muscle and adipose tissue).

A large volume of literature indicates that the brain is implicated in the pathogenies of T2D and/or obesity (see the review Ref. [16]). The first detailed example of central regulation of diabetic hyperglycemia should be leptin, which is a circulating hormone secreted by adipose tissues, sending nutrient signals to the hypothalamus, as well as the nucleus of solitary tract (NTS) and the lateral parabrachial nucleus (LPBN) in the brain [34–38]. Several lines of studies have shown that central infusion of leptin is able to normalize hyperglycemia in rodents with severe insulin-deficient diabetes for couples of days, and this ameliorative effect is achieved through an insulin-independent increase of glucose disposal [39–42]. Insulin-independent manner of glucose regulation refers to increased blood glucose levels to promotes its own disposal, independent of insulin action [27]. Importantly, insulin-independent glucose disposal is comparable to that of insulin action on glucose homeostasis [27]. Most recently, we have demonstrated that activation of leptin receptors (LepRs)-expressing neurons (LepRs^ARH^) in the arcuate nucleus of hypothalamus (ARH) is the neural basis for type 1 diabetes (T1D) hyperglycemia, and the glucose-lowering effect of leptin is largely mediated through the inhibition of LepRs^ARH^ neurons [43]. Furthermore, the impaired nutrient sensing and signs of cellular energy deprivation of LepRs^ARH^ neurons in T1D can be restored by leptin [43]. Overall, these studies clearly support the concept that the brain is capable of normalizing diabetic hyperglycemia via an insulin-independent mechanism. However, a recent study led by Papazoglou and colleagues reveals that activation of oxytocin-expressing neurons in the paraventricular nucleus of the hypothalamus (PVH) causes hyperglycemia via rapid suppression of insulin, while silencing of these neurons induces hypoglycemia through elevation of insulin levels [44].

This framework of central regulation of whole-body glucose homeostasis has been significantly reshaped through the studies investigating the antidiabetic action of fibroblast growth factor (FGF) family of peptides including FGF1, FGF19 and FGF21 [45–49]. FGF19 and FGF21, secreted mainly by the liver and gastrointestinal tract, respectively, exert a transient glucose-lowering action in rodent models of T2D, largely via the brain, and the action is independent of insulin [46, 47]. FGF1, also known as FGF acidic, is produced by multiple cell types in the body including the brain [50]. Recent studies reveal that a single intracerebroventricular (i.c.v) injection of FGF1, at a dose that is ineffective when administered systemically, normalizes hyperglycemia in different rodent models of T2D [48, 49, 51, 52]. More surprisingly, the diabetic remission induced by a single dose of FGF1 has been observed in various models of T2D that can sustain up to four months [48]. Intriguingly, the antidiabetic action of FGF1 is largely mediated by the mediobasal hypothalamus (MBH), where non-neuronal cell types (i.e., tanycytes and astrocytes) represent the major targets [53]. It should be also noted that FGF1 exerts protective effect on insulin secretion and β-cells mass [51, 54]. Nevertheless, FGF1 does not affect blood glucose level in otherwise normal, nondiabetic animals [48]. The authors later speculate that FGF1 causes the brain to “reset” the defended level of blood glucose within a normal range, instead of lowering blood glucose directly [16, 20]. Indeed, Deem and colleagues put forward the notion that there is a possibility that dysfunction of the brain glucoregulatory system contributes to the pathogenesis of diabetes by raising the defended level of glycemia [16, 17]. Of note, clinical studies also report the dysfunction of glucose-regulatory system in the brain in people with diabetes or obesity (see review Ref. [19]).

Therefore, a fundamental question that needs to be carefully addressed is how the brain senses, integrates and responds to the changes in circulating glucose levels. Further, how soon the brain integrates afferent glucose-sensing signals and mounts efferent glucoregulatory responses to restore euglycemia requires further investigations. Concept of glucose-sensing and associated ionic mechanisms need to be discussed before answering these questions.

## Glucose-sensing neurons in the brain

Although all neurons need glucose as a basic fuel source for neuronal viability and physiological function, not all neurons rapidly change their activities in response to glucose fluctuations, a feature named glucose-sensing. It has been well recognized that, in addition to being an energy source, glucose also serves as a nutrient signal that can be detected by glucose-sensors in both the brain and peripheral tissues. Generally, glucose-sensing neurons can be broadly classified into two categories: glucose-excited (GE) neurons, which are activated by an increase of extracellular glucose levels, and glucose-inhibited (GI) neurons, which increase firing frequency and membrane potential in response to a decrease of extracellular glucose concentrations or glucopenia—induced by 2-deoxy-D-glucose (2-DG) [55, 56]. Glucose-sensing neurons are enriched in multiple brain regions including the ARH, the PVH, the VMH, the LH, the supraoptic nucleus, NTS and the area postrema (AP) [30, 57–61]. These sites in the brain, coupled with peripheral glucose sensors form an integrated centre to monitor and/or regulate the circulating glucose levels [11]. However, the ambient glucose levels of these glucose-sensing neurons in each specific brain region might be different. For instance, glucose-sensing neurons in the AP and probably the NTS, are able to detect a higher circulating glucose level as they own a permeable BBB, glucose-sensing neurons in the PVH may not detect circulating glucose level directly as these neurons sit behind the BBB. Interestingly, most neurons in these aforementioned regions employ similar glucose-sensing molecular machinery as are validated in pancreatic β-cells [62, 63]. Glucose-responsive and glucose-sensing neurons are sometimes used interchangeably in the literature. To be precise, glucose-sensing neurons, as introduced above, change their firing activity and membrane potential via sensing ambient glucose fluctuations directly. Glucose-responsive neurons broadly refer to neurons in a glucose-responsive circuit, including glucose-sensing neurons, as well as those downstream neurons that receive projections from glucose-sensing neurons.

Located in the MBH, the VMH is a bilateral cell group with an elliptical shape sitting above the median eminence-ARH [2] (Fig. 1). In addition to thermogenesis, appetitive, social and sexual behaviours, the VMH has also been well-documented in the regulation of body weight and glucose homeostasis [2, 14, 64, 65]. Hetherington and Ranson have demonstrated that lesion of the VMH results into a massive obesity and marked adiposity in rats, and another study revealed that VMH lesions blunt CRR to insulin-induced hypoglycemia [13, 66]. A vast majority of neurons in the VMH are proved glucose-sensing, excited by high glucose level (i.e., GE neurons) or inhibited by high glucose (i.e., GI neurons) [30]. In rat, 14% of VMH neurons are GE neurons, and 3% are GI neurons [67]. An additional 14% are presynaptically excited by decreased glucose (PED neurons), and another 19% presynaptically respond to an increased extracellular glucose [67]. Another study conducted in mice reported that 15–60% of are GE neurons or PED neurons, and only 2% to 7% are GI neurons or PED neurons [68]. Our recent studies revealed that 47% of the dorsomedial subdivision of VMH (dmVMH) neurons and 49% of the central subdivision of VMH (cVMH) neurons, labeled by the steroidogenic factor 1 (SF1), are glucose-sensing [69]. While all estrogen receptor-α (ERα) neurons in the ventrolateral subdivision of VMH (vlVMH) are glucose-sensing, 57% are GE neurons, and 43% are GI neurons [69]. Species difference and heterogeneity of recording protocols, etc. might result in discrepancy upon the validated GE vs. GI neurons distribution in the VMH.

2-DG is a glucose metabolism antagonist, which inhibits glycolysis due to formation and intracellular accumulation of 2-DG-6-phosphate [70]. 2-DG-6-phosphate cannot undergo isomerization to fructose-6-P because it is missing the 2-hydroxyl group [70]. A shortage of glucose caused by 2-DG would trigger CRR in the body to increase blood glucose. The hyperglycemia induced by 2-DG is called glucopenia in this setting. Local glucopenia induced by infusion of 2-DG into rat VMH significantly increases glucagon levels in the circulation, associated with elevated blood glucose [71], whereas infusions of glucose directly into the VMH blocks glucagon release despite of the systemic hypoglycemia [72]. Mice with genetic loss of glutamatergic neurotransmission only in the VMH neurons display impaired responses to hypoglycemia [73]. UCP2-dependent mitochondrial fission in VMH neurons has been reported to mediate glucose-induced neuronal activation and therefore regulates the whole-body glucose metabolism [74]. Abundant neurons in the VMH express glucokinase (GcK), and activation of these neurons increases blood glucose in mice [75]; deletion of GcK reduces hypoglycemia-induced glucagon secretion [76]. Furthermore, neurons in the dmVMH and those in the cVMH receive neuronal inputs from glucose-sensing neurons in the LPBN to defend against hypoglycemia [77, 78]. Our recent studies reveal that all ERα-expressing neurons in the vlVMH are able to sense glucose fluctuations, and prevent mice from severe hypoglycemia [69]. Taken together, all these findings strongly support an essential role of glucose-sensing neurons in the VMH in whole-body glucose homeostasis.

## Glucoregulatory role of non-neuronal cells and perineuronal nets

Most studies investigating glucose-sensing mechanisms in the VMH concentrate on neurons, and yet emerging evidence suggests that non-neuronal cell types (e.g. glial cells and tanycytes), are also of critical importance for glucose-sensing, thereby for the whole-body glucose homeostasis [79, 80].

Hypothalamic glial cells express glucose transporters 2 (GLUT2), a protein involved in glucose-sensing [81]. Marty and colleagues have demonstrated that mice lacking the endogenous *Glut2* gene in glial cells fail to increase plasma glucagon in response to glucoprivation induced either by intraperitoneal or i.c.v. 2-DG injections [23]. However, secretion of plasma glucagon can be restored when *Glut2* is re-expressed in glial cells, not in neurons [23].

Hypothalamic tanycytes are nutrient-sensing cells that are located on the basal walls of the third ventricle of the hypothalamus [79]. Tanycytes can be classified into four different types (i.e., α1, α2, β1, and β2) according to their distribution in the hypothalamic wall [82]. Their cell bodies own direct contact with cerebrospinal fluid (CSF), and extend into the periventricular and lateral parts of the VMH, as well as the ARH [83]. Therefore, they are able to sample glucose levels in the CSF and also circulating glucose levels, thereby relaying this information to glucose-responsive neurons in the VMH or ARH [79, 80, 83]. Tanycytes express GLUT2 and Gck, enabling direct detection of glucose from CSF [81]. Moreover, tanycytes also express monocarboxylate transporters (MCTs), which allow them to shuttle metabolic substrates (e.g., lactate) to the nearby neurons in the VMH [84]. Subsequent studies revealed that inhibition of GLUT2, GcK or MCT of hypothalamic tanycytes impairs neuronal response to fasting and glucose treatment [85–87]. Rohrbach and colleagues have shown that ablation of GcK-expressing tanycytes along the third ventricle results in a low glucagon secretion in response to insulin and 2-DG administration, and induces hyperadiposity via enhancement of food intake [88].

As discussed previously, non-neuronal cell types in the MBH are involved in FGF1-induced diabetic remission [48, 53]. Interestingly, non-neuronal cell types respond more robustly than neurons to i.c.v. injection of FGF1. More specifically, tanycytes and ependymal cells are the most responsive cell types at an early stage, while astrocytes and oligodendrocyte lineage cells become more responsive at later time points. Pioneering work led by Lhomme and colleagues has demonstrated that tanycytes serve as the source of the metabolic signal (i.e., lactate) required by pro-opiomelanocortin (POMC) neurons for hypothalamic glucose-sensing and the control of energy homeostasis [89]. Indeed, a large body of evidence suggests that lactate, produced by astrocytes, is the main fuel source for neurons, particularly when these neurons are activated (see the review [90]). These findings suggest that glial cells in the hypothalamus are involved in glucose-sensing and whole-body glucose homeostasis. However, further studies are warranted to decipher the mechanisms of the interaction between glial cells and neurons mediating the whole-body glucose homeostasis, particularly in the scenarios of diabetes and obesity.

Perineuronal nets (PNNs) are distinct glycosaminoglycan-rich extracellular matrix structures in the brain including those in the MBH [91]. Generally, PNNs enmesh neurons in defined circuit and function as a physical barrier (e.g., ion buffering, physical protection), which also participate in signal transduction, and neuronal activity/plasticity [91]. Work from Alonge and colleagues has illustrated that PNN abundance in the ARH is significantly decreased in obese Zucker diabetic fatty (ZDF) rats, a model of T2D [92]. In addition, the changes of PNNs in ZDF rat model can be reversed by i.c.v. injection of FGF1 at a dose that can induce diabetic remission [92]. Further evidence suggests that enzymatic digestion of PNNs in the ARH impair diabetic remission of central action of FGF1 [92]. Interestingly, reduced PNNs in the ARH are also observed in postnatal ob/ob mice deficient in the leptin gene, which can be restored by leptin during the critical period [93]. Our recent studies indicate that endogenous gonadal hormones are indispensable to maintain normal PNNs in the ARH in both male and female mice [94]. PNNs are also expressed in other brain regions (e.g., VMH), but their function remains largely unknown. Thus, studies are warranted to further characterize the distinct functions of PNNs in whole-body glucose homeostasis.

## Ionic mechanisms of GE and GI neurons in the VMH

**Fig. 2:**
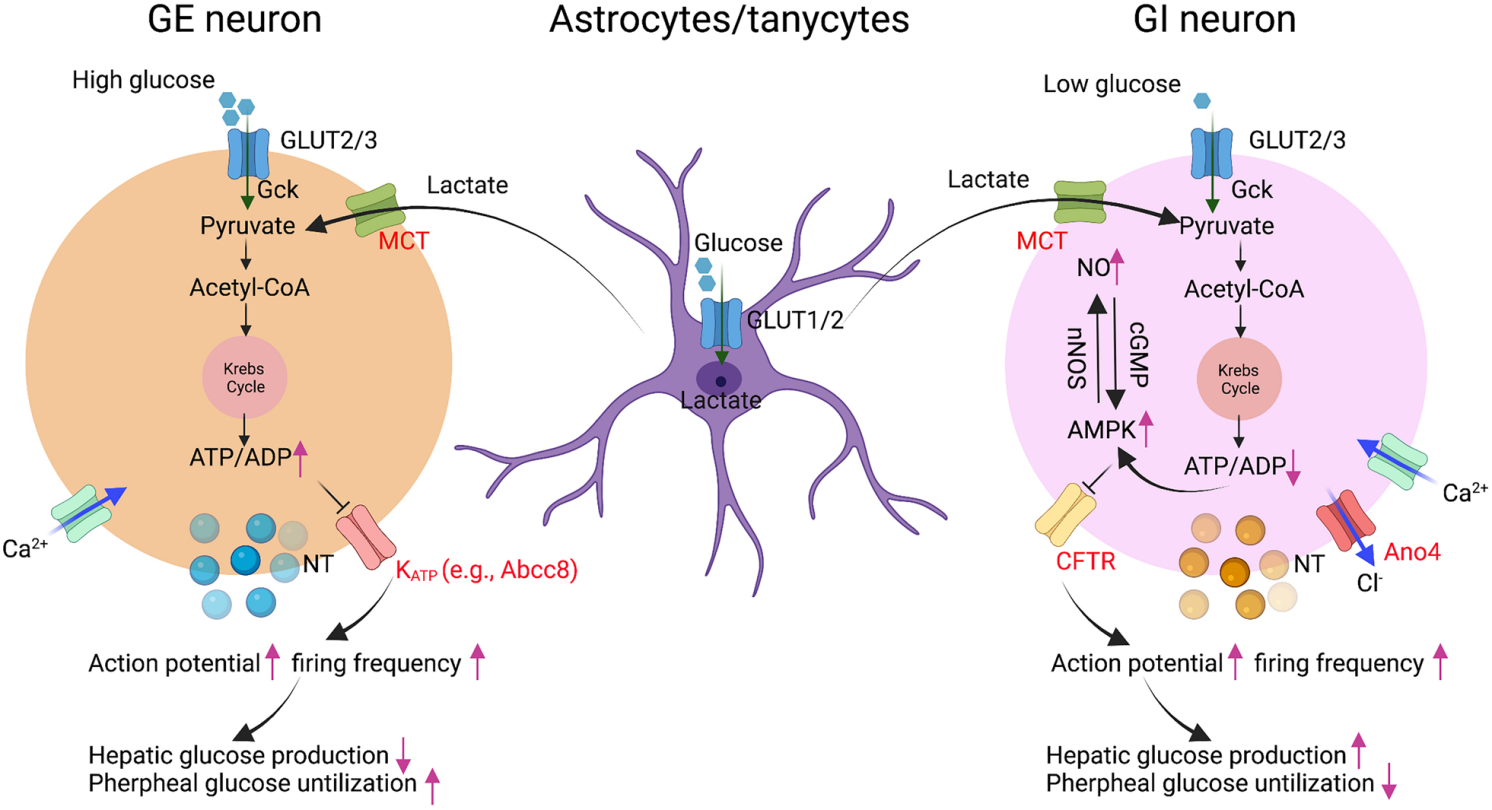
Proposed model of glucose-sensing mechanisms in the VMH. Glucose-sensing neurons can be classified into glucose-excited neurons (GE) and glucose-inhibited neurons (GI) in the VMH. GE neurons is activated by a high glucose (i.e., 5 mM), and GI neurons is activated in response to a low glucose (i.e., 1 mM). For GE neurons, it has been postulated that high glucose leads to an increase of the ATP-ADP ratio and the closure of the K_ATP_ channels (e.g., Abcc8), which leads to plasma membrane depolarization (i.e., increased action potential and firing frequency), and Ca^2+^ entry through voltage-gated channels, thereby increasing neuronal activity and neurotransmitter secretion. Activation of VMH GE neurons results in a reduction in hepatic glucose production and increased peripheral glucose utilization. The ionic mechanism by which GI neurons responds to a low glucose is still not fully understood. A reduction in ambient glucose leads to a decrease of the ATP-ADP ratio and induces an increase of AMP-activated protein kinase (AMPK), which triggers the production of nitric oxide (NO) via neuronal NO synthase (NOS). Activation of cyclic guanosine monophosphate (cGMP) by NO further stimulates AMPK, which induces the closure of chloride channel via cystic fibrosis transmembrane conductance regulator (CFTR), leading to membrane depolarization (i.e., increased action potential and firing frequency) of GI neurons. Meanwhile, anoctamin 4 (Ano4), which is a chloride channel, is opened during hypoglycaemia. Activation of VMH GI neurons leads to an increased in hepatic glucose production and decreased peripheral glucose utilization. Activation of GE or GI neurons by a high or low glucose will be able to trigger the release of relevant neurotransmitters from these neurons to influence downstream targets within glucoregulatory circuity in the brain. Astrocytes/tanycytes also exert glucose-sensing property in the brain. Glucose once taken up by hypothalamic astrocytes or tanycytes via glucose transporters 1/2 (GLUT1/2), it is catabolized into lactate, then exported via monocarboxylate transporters (MCT) to adjacent neurons, altering the formation of pyruvate in neurons. Gck, glucokinase. NT, neurotransmitter. The figure is created in BioRender.com

In vitro studies using brain sections have demonstrated that low glucose (e.g., 1 mM glucose) inhibits GE neurons. By contrast, it activates GI neurons. High glucose (e.g., 5 mM) acts in a totally opposite manner. A fundamental question in the field of glucose-sensing is what ion channels mediate the totally opposite electric responses in GE vs. GI neurons when subjected to either low or high glucose. In terms of ionic mechanisms of GE neurons, high glucose leads to an increase of the ATP-ADP ratio and the closure of the K_ATP_ channels, which results in plasma membrane depolarization and Ca^2+^ entry through voltage-gated channels, thereby increasing neuronal activity and neurotransmitter secretion [55, 95–97] (Fig. 2). Interestingly, the K_ATP_ channel has been reported to mediate GE neurons response in multiple brain regions [55, 95–97]. On the other hand, for GI neurons, the mechanisms related a low glucose level to augmented firing activity is less clear, but it is considered to be lined with a tandem-pore K^+^ channel and/or closure of the cystic fibrosis transmembrane conductance regulator (CFTR) that is a chloride channel (see the review Ref. [14]) (Fig. 2).

A recent study led by Quenneville et al. reported that AMP-activated protein kinase (AMPK) is required for GI neurons activity in the VMH by controlling the expression of antioxidant enzyme Txn2, but its loss of function does not affect glucose-sensing capacity of GE neurons [98] (Fig. 2). It has been widely accepted that AMPK is an intracellular energy sensor that is activated in conditions of low energy, leading to promote energy production and reduce energy waste [99]. Recent evidence highlights the critical role of AMPK in the VMH for thermogenesis (for the review see Ref. [65]). Deletion of AMPKα1 in SF1 neuron in the VMH (VMH^SF1^) reverses high-fat diet-induced obesity via increasing thermogenesis of brown adipose tissues [100]. Soon after this observation, the authors have developed small extracellular vesicles, a carrier of plasmids encoding a dominant negative AMPK α1 mutant that can be used to target VMH^SF1^ neurons to reduce body weight of obese mice [101]. It is still however elusive whether the effect of AMPK on GI neurons is related to actions of AMPK on feeding, energy expenditure, glucose and lipid homeostasis. There is also evidence implicating that neuronal nitric oxide synthase 1 (NOS1) is necessary for appropriate glucose detection by GI neurons in the VMH, and low glucose activates NOS1 and increases NO production in mediobasal hypothalamus [102] (Fig. 2). Furthermore, activation of NOS1 neurons (VMH^NOS1^) in the VMH elicits hyperglycaemia, indicating that a large majority of VMH^NOS1^ appears to be GI neurons, although NOS1 neurons are a subset of VMH^SF1^ neurons [103]. In combination with whole-cell patch clamp and RNA-sequencing, we have identified that hypoglycemia activates GI-ERα^vlVMH^ neurons through the opening of anoctamin 4 (Ano4, a chloride channel), and inhibits GE-ERα^vlVMH^ neurons through the opening of the Abcc8-containing K_ATP_ channel [69]. Interestingly, large-scale human exome sequencing further revealed that variant of *ANO4* gene is associated with human obesity, and perhaps glucose dysregulation [104]. Nonetheless, more investigations are warranted to elucidate the ionic mechanisms by which GE vs. GI neurons sense ambient glucose fluctuations, thereby inducing opposite electrical responses.

Emerging evidence also implicates a significant role of non-neuronal cells in glucose sensing in the brain. Early work from Magistretti and colleagues has shown that there is a metabolic coupling between neurons and astrocytes for glucose usage and energy metabolism [105] (Fig. 2). It has been well documented that glucose once taken up by hypothalamic tanycytes via glucose transporters 1/2 (GLUT1/2), would be catabolized into lactate, then exported via MCT to nearby neurons, where it is oxidized [84] (Fig. 2). However, indeed, the nature of the interaction between neurons and these non-neuronal cells is still not fully understood, which necessitates further studies.

## How does the brain regulate circulating glucose?

**Fig. 3:**
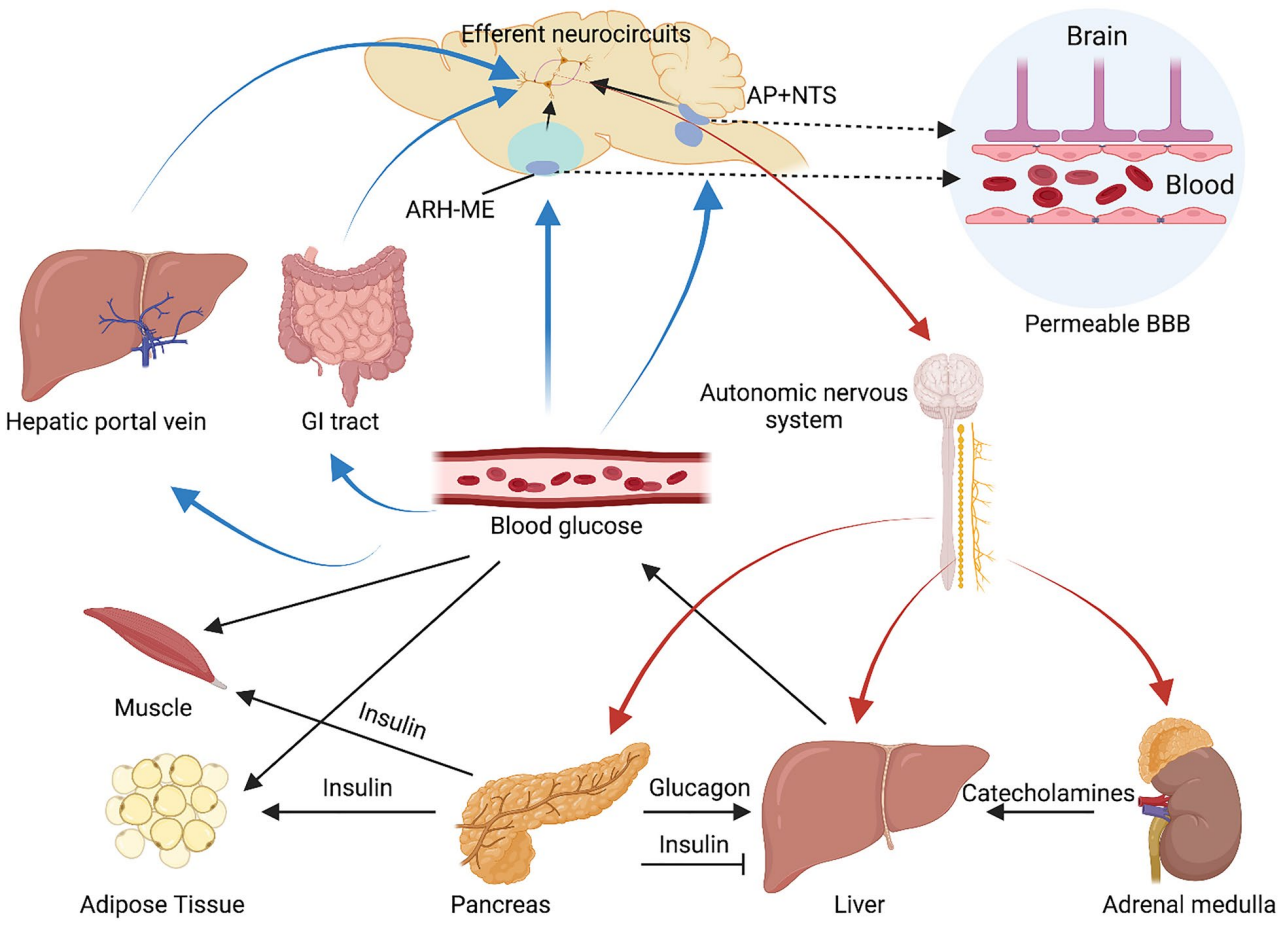
Central regulation of glucose homeostasis. Changes of the blood glucose level (i.e., hypoglycemia or hyperglycemia) is detected by peripheral glucose sensors in hepatic portal vein and gastrointestinal tract (GI tract), as well as glucose-sensing neurons in the acute nucleus of hypothalamus-median eminence (ARH-ME), and area postrema (AP) and nucleus of the solitary (NTS) in the hindbrain (blue arrows), where the blood brain barrier (BBB) is permeable. Then these afferent signals are transmitted to the ventromedial hypothalamic nucleus (VMH) or other glucose-responsive regions in the brain (black arrows in the brain) where they are integrated to mount the efferent responses to restore euglycemia. Maintenance of blood glucose level is achieved by balancing entry of glucose into blood vessels, and glucose uptake by peripheral tissues (e.g., adipose tissue and muscle). Insulin is secreted from pancreatic β-cells in response to a high circulating glucose level, which promotes glucose uptake into adipose tissue and muscle, and inhibits glucose production in the liver (black arrows) via suppression of glucagon from α-cells. In the context of hypoglycemia, counterregulatory hormones—glucagon from pancreatic α-cells and catecholamines from adrenal gland are released to promote hepatic glucose production, thereby increasing glucose entry into the blood vessels. At the same time, glucose removal from blood vessels via glucose uptake is inhibited. The brain is able to balance glucose entry and uptake via autonomic nervous system (both sympathetic and parasympathetic nervous system) to reset/restore the blood glucose level to a normal range (red arrows). The figure is created in BioRender.com

Under physiological conditions, rising blood glucose stimulates pancreatic β-cells to secrete insulin. Secreted insulin then inhibits hepatic glucose production, and increases glucose uptake in adipose tissues and skeletal muscles. Meanwhile, insulin also inhibits glucagon secretion, which is secreted from α-cells to stimulate hepatic glucose production. Indeed, insulin and glucagon secretion is also influenced by both sympathetic and parasympathetic branches of autonomic nervous system under a variety of conditions. With cell bodies positioned in the celiac ganglia and superior mesenteric ganglia, sympathetic efferent fibres in the islet releases norepinephrine to stimulate glucagon secretion via binding to α-adrenergic receptors on pancreatic α-cells; inhibits insulin secretion via activation of β-adrenergic receptors of β-cells. Originating from the dorsal motor nucleus of the vagus in the hinderbrain, the parasympathetic fibres sends cholinergic inputs to the islet to stimulate glucagon secretion on α-cells, as well as increase insulin secretion via release of acetylcholine binding to the muscarinic receptors on β-cells. In addition to the islet, the brain also modulates the release of epinephrine from the adrenal medulla [21] (Fig. 3).

Glucose is the primary fuel for the brain, and glucose levels in the brain therefore need to be finely regulated and maintained at a constant level. The entry of glucose into the brain is mainly mediated by glucose transporter 1/2 (GLUT1/2) that is expressed in astrocytes and endothelial cells of the BBB, as well as in tanycytes along the CSF barrier [106]. Glucose levels in the brain interstitial fluid (ISF) are maintained at only 20–30% of the circulating blood level, ranging from 1–2.5 mmol/l at baseline [107–109]. This applies to the hypothalamus and other brain regions where a large number of glucose-sensing neurons are enriched. Yet there are glucose-sensing neurons identified in the brain that are distributed within or in close proximity to circumventricular organs (CVOs), where permeable BBB is present [22]. This could be the case for neurons in the NTS and AP in the hindbrain, as well as the ARH and adjacent median eminence, in which neurons with glucose-sensing capacity are exposed to a higher blood level than it in the brain ISF (Fig. 3).

It should be noteworthy that glucose levels in the brain ISF are relatively stable under most if not all physiological conditions [110, 111]. A drop of the circulating blood glucose (e.g., insulin-induced hypoglycemia) is reflected in a way that is dampened and delayed in the brain ISF [22]. In other words, the ISF glucose level does not represent the temporal dynamics of the glucose in circulation during insulin-induced hypoglycemia. However, the brain, particularly the VMH, is initially involved in CRR to insulin-induced hypoglycemia (Fig. 3). Then how does the brain know immediately after there is a sharp drop of the circulatory blood glucose and then instigate CRR to restore euglycemia? ISF brain glucose levels are stable during the initial stage of insulin-induced hypoglycemia, indicating that the afferent signals of changes in the circulating blood glucose should not be derived from those glucose-sensing neurons, including those in the VMH. Indeed, there is evidence that the intrinsic glucose-sensing capacity of GI neurons in the VMH is not involved in the physiological responses mounted to hypoglycemia, but rather represents a fail-safe system in case of failure of peripheral glucose sensors detecting hypoglycemia [98]. Bentsen and colleagues suggest that central control of blood glucose highly relies upon the capacity to sense the circulating glucose level, rather than glucose level in the brain ISF sensed by local glucose-sensing neurons [22]. Glucose-sensing neurons in the brain will be able to impact whole-body glucose homeostasis only under pathological conditions when brain ISF glucose would be impacted [22]. As such, these glucose-sensing neurons in the brain severs as ‘fail-safe’ mechanisms for responding to pathological fluctuations of the brain ISF glucose when physiological defense fails or have been overridden [20, 22]. If this is true, then what afferent limbs of glucoregulatory circuit transmit to the brain, and how? What is the significance of glucose-sensing neurons in the VMH as they do not sense perturbations in the circulating glucose level because they sit behind BBB anatomically? Are glucose-sensing neurons in the VMH effector neurons constituting the efferent limb of glucoregulatory system? Yet there are still no definitive answers to these questions. More exhaustive studies are thus required to further investigate the significance of brain in whole-body glucose homeostasis, particularly under pathological conditions (e.g., diabetes and obesity).

## Heterogeneity of VMH neurons in glucose regulation

Anatomically, the VMH contains a core region and a cell-poor shell region. The VMH core region is comprised of three subdivisions: dmVMH, cVMH and vlVMH. It should be noted that the VMH shell region also contains a small number of neurons appearing as a ring that surrounded the core region [112]. Most of cells in the core region are glutamatergic, with few are GABAergic [113]. On the other hand, GABAergic neurons are predominant in the shell region, which are thought to send inhibitory outputs onto neurons in the core [112]. Mice lacking *Vglut2* in the VMH^SF1^ neurons results in a lower fasting glucose, and impairs CRR to insulin-induced hypoglycemia and 2-DG-induced glucopenia [73]. This suggests that glutamate released by SF1 neurons is of critical importance to prevent hypoglycemia [73]. Chan and colleagues have demonstrated that pharmacological blockade of GABA receptors in the VMH stimulates glucagon and epinephrine secretion, without affecting blood corticosterone in response to hypoglycemia [114]. In a following study, the authors further revealed that recurrent hypoglycemia enhances GABAergic inhibitory tone in the VMH, which contributes to the impairment of CRR (i.e., reduction in glucagon and epinephrine release) to subsequent bouts of acute hypoglycemia [115].

### SF1 neurons

**Fig. 4:**
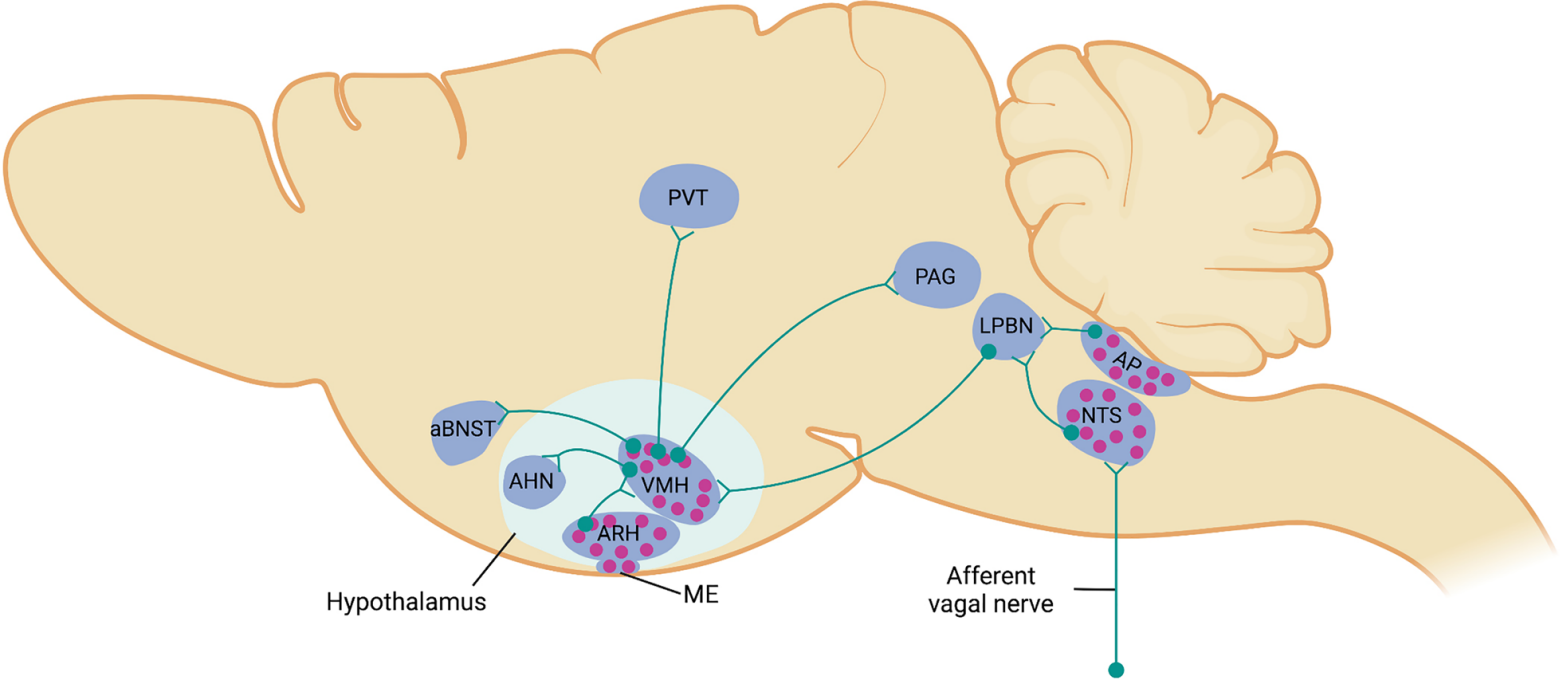
Neurocircuitry of VMH neurons in glucose homeostasis and other homeostatic responses. Optogenetic stimulation of VMH neurons induces hyperglycemia via projections from SF1 neurons to the anterior bed nucleus of the stria terminalis (aBNST), as well as projections from NOS1 neurons to aBNST and the periaqueductal gray (PAG). Glucose-sensing neurons are enriched in the ARH-ME and brainstem, i.e., area postrema (AP) and nucleus of solitary tract (NTS), such that these neurons can detect changes in the circulating glucose level directly. Glucose-sensing neurons in the ARH-ME send projections to the VMH. Meanwhile, neurons in the VMH also receive efferent inputs from the lateral parabrachial nucleus (LPBN), where it may receive projections from AP and NTS regarding changes of circulating glucose level. There is a possibility that NTS neurons might also receive inputs from glucose sensors in the gastrointestinal gut via afferent vagal nerve. It is still under debate whether glucose-sensing neurons in the VMH can sense the blood glucose fluctuations directly before efferent responses are mounted. For other homeostatic responses, activation of SF1 neurons is also involved in suppression of food intake via projection to the paraventricular thalamus (PVT). SF1 neurons projection to the PAG induces inflexible immobility, and to anterior hypothalamic nucleus (AHN) pathway promotes avoidance. Red dots represent glucose-sensing neurons. The figure is created in BioRender.com

A vast majority of neurons in the dmVMH and cVMH can be exclusively labeled by a transcription factor SF1 [116, 117]. Early studies revealed that *SF1* gene knockout mice are obese and display abnormal VMH development, in favor of the notion that SF1 neurons in the VMH are critically important for body weight control [111, 116]. Subsequent studies have shown that activation of VMH^SF1^ neurons is required for CRR to insulin-induced hypoglycemia. Optogenetic stimulation of VMH^SF1^ neurons induces hyperglycemia in otherwise normal mice via projections to the anterior part of bed nucleus of the stria terminalis (BNST), while receiving efferent inputs from the LPBN [78] (Fig. 4). By contrast, another study has demonstrated that activation of VMH^SF1^ neurons with designer receptors exclusively activated by designer drugs (DREADD) does not induce significant hyperglycemia, but a lower blood glucose in the context of both glucose tolerance and insulin tolerance tests [118]. Meanwhile, it is suggested that inhibition of VMH^SF1^ neurons via DREADD worsen glucose tolerance [74]. However, another study led by Zhang et al. showed that activation of VMH^SF1^ neurons via DREADD indeed induces hyperglycemia, impairs glucose tolerance and reduces insulin sensitivity, although this activation also inhibits food intake via projection to the paraventricular thalamus (PVT) [119] (Fig. 4). The discrepancy among these studies is still unknown, but it is more likely to ascribe to the different clusters of neurons that might be targeted in these studies, given that a high neuronal heterogeneity within SF1 neurons [2, 14]. However, it should be noted that glucose alteration was triggered by a very high optogenetic stimulation (40 Hz for 1 h), that is likely beyond the spontaneous firing frequency of neurons in the VMH [78]. In addition, other studies reported that optogenetic stimulation of VMH^SF1^ neurons is associated with defensive/avoidance, freezing, jumping and escaping behaviors [120, 121] (Fig. 4). It is still unknown whether alteration of such behaviors via optogenetic stimulation would affect glucose homeostasis. One potential argument is that the high optogenetic stimulation at 40 Hz, which is not typically observed in SF1 neurons in vitro and in vivo, inherently induces stress-behaviors, which may artificially affect glucose balance.

Since the generation of SF1-Cre transgenic mouse line, an increasing number of studies have been conducted to examine the significance of a variety of molecules or neurotransmitters (e.g., BBsome, Rap1, gGluR5, AMPKα, CB1R, Socs3, STAT3, prostaglandin, etc.) based on SF1 neurons within the VMH regarding glucose metabolism, energy homeostasis and body weight control [98, 100, 122–128]. Technically, generation of SF1-Cre mouse line enables ablation of general factors or targets by crossing with specific flox/flox mouse line. Recently, Fosch and colleagues have summarized key molecules or targets implicated into glucose homeostasis and/or energy balance in the VMH based on SF1-Cre transgenic mice (for the review see Ref. [129]). In addition, most recent studies suggested that Rap1 and prostaglandin in the VMH have also been implicated into glucose homeostasis [123, 128].

Encoded by *Rap1a* and *Rap1b*, Rap1 is a small GTPase that is expressed throughout the whole body including the brain [130]. We have shown that increasing Rap1 activity in VMH^SF1^ neurons elevates blood glucose without alteration of body weight; ablation of Rap1 in VMH^SF1^ neurons remarkedly lowers blood glucose, improves both glucose tolerance and insulin sensitivity in high fat diet (HFD)-fed mice [123]. Work from Lee and colleagues has demonstrated that a glucose injection elicits a reduction in arachidonic-acid-containing phospholipids concomitant with an increase of prostaglandin production derived from phospholipids in the VMH [128]. A lower production of prostaglandin via knockdown of cytosolic phospholipase A2 (cPLA2) in SF1 neurons in the VMH increases peripheral glucose metabolism during regular chow diet [128]. By contrast, a reduced production of prostaglandin via knockdown of cPLA2 in the VMH during HFD feeding impairs hepatic insulin sensitivity, suggesting that the role of prostaglandin regarding glucose metabolism is differential in HFD and chow diet [128].

### Glucokinase-expressing neurons

It has been long known that GcK, as a glucose sensor, plays a crucial role in glucose homeostasis [131, 132]. Once transported inside the cell via GLUT2/3, glucose is phosphorylated to glucose-6-phosphate by GcK, which is a rate-limiting step in glycolysis [131]. Mutation of *GcK* gene results in maturity onset diabetes of the young, and persistent hyperinsulinemic hypoglycemia of infancy [133, 134]. The role of GcK in central glucose sensing and whole-body glucose homeostasis was unknown due to its low expression in the brain. In 2013, Jeffrey Friedman group generated a GcK-Cre transgenic mouse line with neuronal/endocrine-specific Gck promoter driving *Cre* expression; GcK positive neurons therefore can be visualized and mapped throughout the brain via crossing with a Cre-reporter mouse line [135]. The VMH is one of the regions in the hypothalamus where GcK-expressing cells are densely populated [135]. More importantly, GcK-expressing cells in the VMH can be activated by glucose or 2-DG, [135]. Later studies conducted by the same group further revealed that activation of GcK-expressing neurons in the VMH via remote radio waves or magnetic field increases blood glucose and stimulates feeding [75]. By contrast, inhibition of GcK-expressing neurons in the VMH significantly reduces blood glucose, and impairs CRR to 2-DG-indcued glucopenia [75]. These studies suggest that GcK neurons are more likely GI neurons. In addition, Steinbusch and colleagues showed that ablation of *GcK* gene in VMH^SF1^ neurons impairs glucagon secretion in response to hypoglycemia, as well as a reduced parasympathetic and sympathetic nerve activation during 2-DG-induced glucopenia in female mice [76]. Furthermore, in female mice, inactivation of *GcK* gene in VMH^SF1^ neurons increases white fat mass and adipocyte size, and reduces lean mass [76]. However, lack of Gck expression does not alter glucose-sensing properties of both GE and GI neurons among SF1 neurons [76].

### Leptin receptors-expressing neurons

LepRs are abundantly expressed in the dmVMH, and a majority of LepRs neurons also express SF1 [117, 136]. However, activation of LepRs-expressing neurons in the VMH does not alter blood glucose level, indicating that those SF1 neurons that do not express LepRs are responsible for hyperglycemic effects during optogenetic stimulation of VMH^SF1^ neurons [78]. However, LepRs in the VMH are indispensable for normal energy homeostasis, and knockout of LepRs in VMH^SF1^ neurons results in obesity and accumulation of fat store by decreasing energy expenditure [117]. Moreover, LepRs in the VMH mediate leptin effect on increasing glucose disposal into skeletal muscle and brown adipose tissue [117, 137, 138].

### BDNF neurons

The VMH also contains a large number of neurons expressing brain-derived neurotrophic factor (BDNF) that are associated with regulation of energy balance [139]. More specifically, i.c.v. administration of BDNF suppresses food intake via inducing neuronal activity in the VMH, as well as other hypothalamic nuclei [140]. Mice with deletion of VMH-derived BDNF results in hyperphagia and body weight again, concomitant with hyperglycemia, hyperleptinemia and hyperinsulinemia [140]. Maekawa and colleagues revealed that reduction of BDNF in the VMH contributes to visceral fat accumulation and hyperleptinemia in the type 2 diabetic Goto-Kakizaki rats [141]. Interestingly, a recent study led by Kamitakahara et al. revealed that loss of BDNF in VMH^SF1^ neurons results in increased inhibitory synapse onto SF1 neurons, as well as impaired physiological responses to insulin-induced hypoglycemia, including a reduction in secretion of glucagon [142].

### CCKBR neurons

A portion of VMH neurons also express cholecystokinin receptor B (CCKBR), and optogenetic stimulation of CCKBR-expressing neurons in the VMH (VMH^CCKBR^) promotes hyperglycaemia primarily by increasing circulating catecholamines and glucocorticoids levels, but independent of islet cells – levels of glucagon and insulin are stable during stimulation [143]. In contrast to VMH^SF1^ neurons engaging into energy homeostasis and body weight control, silencing of VMH^CCKBR^ neurons does not perturb energy expenditure or body weight, but inducing hypoglycaemia [117, 143]. Moreover, it also impairs hepatic glucose production, as well as blunts CRR to insulin-induced hypoglycemia and 2-DG-induced glucopenia [117, 143]. Intriguingly, silencing of VMH^CCKBR^ also ameliorates the hyperglycemia and weight loss in mice rendered diabetes induced by streptozotocin (STZ), despite similarly low insulin levels and β-cells mass [143]. Thus, these findings collectively suggest that VMH^CCKBR^ neurons are able to modulate the blood glucose setpoint and maintenance independent of β-cells integrity and islet hormone concentrations. This remarkably reshapes the traditional notion that hyperglycemia associated with diabetes results exclusively from impaired pancreatic islet function (e.g., insulin secretion and/or resistance) in the STZ animal model. The VMH glucoregulatory mechanism is well established for CRR yet not for the diabetic hyperglycaemia. The studies of CCKBR neurons in the VMH is one of the supporting evidence of this concept.

### MC3R neurons

Deletion of melanocortin 3 receptor (MC3R) in mice results in alteration of nutrient partitioning and accumulation of fat over muscle mass [144]. Rescuing *Mc3r* transcription in the VMH improves metabolic control, but does not restore appetite or nutrient partitioning [144]. A recent study led by Sutton et al. revealed that MC3R-expressing VMH neurons (VMH^MC3R^) sense glucose fluctuations through direct and indirect mechanisms [145]. BNST-projecting VMH^MC3R^ neurons integrate excitatory inputs from a variety of brain regions relevant to glucose homeostasis including POMC neurons in the ARH, and neurons in the LPBN [145]. DREADD-mediated activation of VMH^MC3R^ neurons blunts, and silencing enhances glucose excursion in the context of glucose tolerance tests [145]. It remains unknown whether other CRR hormones are also involved, although the glucose disposal effect does not involve insulin. Sex difference exists regarding MC3R regulation of feeding-related reward in the limbic structure, it is however unknown whether it also exist upon glucose homeostasis in the VMH [146, 147].

### PACAP neurons

Pituitary adenylate cyclase-activating peptide (PACAP) is another identified molecule that is implicated in glucose homeostasis in the VMH. Approximately 72% VMH^PACAP^ neurons respond to a lower glucose (2.5 mM to 1 mM) directly with an increasing firing rate, and those responsive neurons are proved to be intrinsic GI neurons [148]. Activation of VMH^PACAP^ neurons via the DREADD strategy causes a delayed increase in baseline glucose levels concomitant with a suppression of insulin release, which is not associated with a change in glucagon levels [148]. Similar to VMH^CCKBR^ neurons, VMH^PACAP^ neurons also integrate inputs from CCK-containing neurons in the LPBN [77, 143, 148]. In addition to glucose regulation, PACAP in the VMH is also involved in feeding regulation. For instance, direct injection of PACAP into the VMH induces hypophagia and thermogenesis; knockout of PACAP in the VMH reduces food intake, and yet overexpression of it enhances food intake [149, 150].

### NOS1 neurons

It has been reported that NO production in the VMH is indispensable for glucose-sensing in GI neurons, as well as CRR to insulin-induced hypoglycemia [102, 151]. Anatomically, VMH^NOS1^ neurons are a subset of VMH^SF1^ neurons. Likewise, optogenetic stimulation of VMH^NOS1^ neurons causes robust hyperglycemia via activation of CRR (i.e., glucagon and corticosterone), without a suppression on insulin release [103]. Moreover, VMH^NOS1^ neurons send projections to the BNST and the periaqueductal gray (PAG) that are implicated in hyperglycemia during optogenetic stimulation [103] (Fig. 4). It should be noteworthy that optogenetic stimulation of VMH^NOS1^ neurons eliciting hyperglycemia is accompanied by freezing behavior [103].

### ERα neurons

Neurons in the VMH expressing ERα are most limited, but not exclusively to the vlVMH, where SF1 is not expressed during adulthood [152]. In addition to glucose regulation, ERα signaling in the VMH also contributes to energy homeostasis (via thermogenesis and physical activity), as well as aggressive sexual behaviors [64, 153]. Moreover, we have previously revealed that female mice lacking ERα in SF1 neurons develop anovulation and infertility, in addition to hypometabolism and abdominal obesity [154]. Our most recent studies have shown that all ERα neurons in the vlVMH are intrinsically glucose-sensing neurons, in which 43% are GE and 57% are GI [69]. Interestingly, a subset of GI-ERα^vlVMH^ neurons preferentially project to the medioposterior ARH (mpARH) and a subset of GE-ERα^vlVMH^ neurons preferentially project to the dorsal Raphe nucleus (DRN), which function in a synergistic manner to prevent severe hypoglycemia when glucose drops [69].

A summary of identified neuronal populations in the VMH that are involved in whole-body glucose homeostasis and other homeostatic responses was shown in Fig. 1.

## Sex differences in the VMH

Accumulating evidence suggests that the VMH displays sex differences in hormone secretion, synaptic organization, gene expression and neuron function [155–157]. As addressed in the previous section, inactivation of *Gck* in VMH^SF1^ neurons results in increased fat mass and adipocyte size, impaired glucagon secretion during hypoglycaemia, and suppression of autonomic nervous activity in response to 2-DG-induced glucopenia only in female mice [76]. Furthermore, Micaella Fagan et al. have demonstrated that depletion of metabotropic glutamate receptor subtype 5 (mGluR5) in SF1 neurons remarkably impairs insulin sensitivity, glucose and lipid balance, and sympathetic output only in female but not male mice [124]. More specifically, the authors further revealed that these sex-specific roles played by mGluR5 arise from the disrupted functional interaction between mGluR5 and estrogen receptors on VMH^SF1^ neurons—reduction in excitability and firing frequency to estrogen in females [124]. Indeed, sex difference in the VMH regarding energy homeostasis and feeding behaviour dates back to our early studies investigating effect of ERα in SF1 neurons. Genetic depletion of ERα in SF1 neurons causes obesity, adiposity and visceral fat distribution concomitant with glucose intolerance only in female mice [154]. Recently, Van Veen et al. defined six neuronal populations in the mouse VMH through single cell RNA sequencing of VMH cells collected 10 day after birth from SF1-Cre reporter mice [158]. The authors further revealed that ERα is enriched in 3 clusters of neurons showing sex-biased expression, which are restricted to the vlVMH in females [158]. Another study involving single cell RNA sequencing of VMH from adult mouse identified 40 neuronal clusters and 6 non-neuronal clusters, in which 17 transcriptomic types of neurons are restricted to the vlVMH, including several sexually dimorphic populations [159]. Indeed, early studies from the Vanessa H Routh group revealed that there is a sex difference with respect to glucose-sensing neurons in the vlVMH [160]. More specifically, GI neurons in the vlVMH from male mice respond more robustly to low glucose (i.e., 0.1 mM) than those from female mice, suggesting those GI neurons are inherently sexually dimorphic [160].

The existence of sex differences in energy balance and glucose homeostasis has been significantly appreciated recently, though the underlying mechanism is still not fully understood [161, 162]. Estrogen/ERα system should be involved in the sex differences among the aforementioned studies since a variety of phenotypes have been observed only in female mice, but not in male mice. In addition to gonadal hormones (e.g., estrogen), sex chromosomes (e.g., Sry and X chromosome dosage) and other factors (e.g., POMC, TAP63, and Sirt1) are also implicated in energy homeostasis differentially in males vs. females (for the review see Ref. [163]). It necessitates further studies on whether sex chromosomes and other factors (not gonadal hormones/receptors) are also involved in the sex differences in the VMH.

## Perspectives and conclusions

It is well established that the VMH is one of the most critical hypothalamic nuclei in the brain for glucose-sensing, as well as whole-body glucose homeostasis. Dysfunction of this homeostasis in the VMH is closely linked with impaired physiological responses to hypoglycemia and glucopenia. Emerging evidence suggests the brain is greatly implicated into pathogenesis of diabetes. Efforts to identify the link between the brain (e.g., the VMH neurons) and diabetes is a priority in this field. There are heterogeneous types of neurons in the VMH, which can be phenotypically labeled (e.g., SF1, CCKBR, NOS1 and ERα), and have been profiled in detail so far. Some of these neurons are intrinsically glucose-sensing, enabling them to sense ambient glucose fluctuations directly. However, growing evidence reminds that other glucose-responsive neurons in the brain are of paramount importance for glucose homeostasis. The significant roles played by non-neuronal cell types, particularly those sitting along the hypothalamic wall, is still at infancy, and thus deserves further studies. It is still not fully understood how the VMH orchestrates whole-body glucose homeostasis under physiological conditions, as well as in pathological scenarios. Although significant advances have been made regarding the brain-body control of glucose homeostasis, the role of the hypothalamus, particularly the VMH, is far more sophisticated. Thus, more exhaustive studies are needed to unravel the associated mechanisms, and ultimately lead to new effective treatments of diabetes.

## Data Availability

Not applicable.

## Abbreviations

2-DG: 2-Deoxy-D-glucose
3 V: Third ventricle
AMPK: AMP-activated protein kinase
Ano4: Anoctamin 4
ARH: Arcuate nucleus of hypothalamus
AP: Area postrema
BNST: Bed nucleus of the stria terminalis
BBB: Blood brain barrier
BDNF: Brain-derived neurotrophic factor
CNS: Central nervous system
CSF: Cerebrospinal fluid
CCKBR: Cholecystokinin receptor B
CVOs: Circumventricular organs
CRR: Counterregulatory response
CFTR: Cystic fibrosis transmembrane conductance regulator
DREADD: Designer receptors exclusively activated by designer drugs
DRN: Dorsal Raphe nucleus
ERα: Estrogen receptor-α
FGF: Fibroblast growth factor
GcK: Glucokinase
GLUT1: Glucose transporters1
GLUT2: Glucose transporters 2
GE: Glucose-excited
GI: Glucose-inhibited
mGluR5: Glutamate receptor subtype 5
HFD: High fat diet
ISF: Interstitial fluid
LH: Lateral hypothalamus
i.c.v: Intracerebroventricular
LPBN: Lateral parabrachial nucleus
LepRs: Leptin receptors
MBH: Mediobasal hypothalamus
MC3R: Melanocortin 3 receptor
MC4R: Melanocortin-4 receptor
MCT: Monocarboxylate transporter
NOS1: Nitric oxide synthase 1
NTS: Nucleus of solitary tract
PVH: Paraventricular nucleus of the hypothalamus
PVT: Paraventricular thalamus
PNNs: Perineuronal nets
PACAP: Pituitary adenylate cyclase-activating peptide
PED: Presynaptically excited
SF1: Steroidogenic factor 1
STZ: Streptozotocin
T1D: Type 1 diabetes
T2D: Type 2 diabetes
VMH: Ventromedial hypothalamic nucleus
ZDF: Zucker diabetic fatty
